# Quality Assessment and Classification of *Codonopsis* Radix Based on Fingerprints and Chemometrics

**DOI:** 10.3390/molecules28135127

**Published:** 2023-06-29

**Authors:** Xuxia Liu, Zhengjun Chen, Xin Wang, Wenrong Luo, Fude Yang

**Affiliations:** 1 School of Pharmacy, Gansu University of Traditional Chinese Medicine, Lanzhou 730013, China288142476@xbmu.edu.cn (X.W.); 2 Gansu Provincial Hospital of Chinese Medicine, Lanzhou 730050, China

**Keywords:** HPLC fingerprint, PCA, O2PLS-DA, marker compounds, quality control

## Abstract

In China, *Codonopsis* Radix (CR) is frequently consumed both as food and medicine. Here, a comprehensive strategy based on fingerprinting and chemometric approaches was created to explore the influence of origins, storage time and kneading processing on the quality of CR. Firstly, high-performance liquid chromatography with diode array detection was used to obtain the fingerprints of 35 batches of CR from six different origins and 33 batches of CR from varying storage times or kneading procedures. Secondly, chemometric methods including similarity analysis (SA), principal component analysis (PCA), hierarchical clustering analysis (HCA), and two-way orthogonal partial least square with discriminant analysis (O2PLS—DA) were used to evaluate the differences of chemical components in CR so as to identify its source and reflect its quality. Moreover, 13 and 16 major compounds were identified as marker compounds for the discrimination of CR from different origins, storage time and kneading processing, respectively. Furthermore, the relative content of the marker components and the exact content of Lobetyolin were measured, indicating that the contents of these components vary significantly between various CR samples. Meanwhile, the chemical components of CR were identified using Mass spectrometry. According to the findings of our investigation, the quality of CR from Gansu was the best, followed by Shanxi and then Sichuan. The quality of CR from Chongqing and Guizhou was poor. At the same time, the quality of CR was the best when it was kneaded and stored for 0 years, indicating that the traditional kneading process of CR is of great significance. Conclusively, HPLC fingerprint in conjunction with chemical pattern recognition and component content determination can be employed to differentiate the raw materials of different CR samples. Additionally, it is also a reliable, comprehensive and prospective method for quality control and evaluation of CR.

## 1. Introduction

As is known, traditional Chinese medicines (TCMs) have been widely used to treat various diseases in China for a long time, some of which are medicine and food homology materials (MFHM). To date, the development of MFHM has a long history [1]. *Codonopsis* Radix (CR) is also a kind of MFHM with unique taste and medicinal value, which can be used as a replacement for expensive ginseng [2]. CR is the dried roots of *Codonopsis pilosula* (Franch.) Nannf. (*C. pilosula*), *Codonopsis pilosula* Nannf. var. modesta (Nannf.) L.T.Shen (*C. pilosula* var. modesta), and *Codonopsis tangshen* Oliv. (*C. tangshen*) [3], which has various medicinal properties, such as antidiabetic [4], gastrointestinal protection [5], immunomodulatory [6], anticancer [7] functions and regulate intestinal function [8]. Because of its good nutritional properties, CR is also an important ingredient that is frequently added to meals and is particularly well-liked in Asian nations [9,10]. A significant producer of CR is China. CR is cultivated in Gansu, Sichuan, Shanxi, Guizhou, Hubei, and Chongqing provinces in China. Among them, the quality of CR in Gansu producing area is best and the output is the largest, accounting for more than 80% of the total production [11,12].

There is no doubt that the ecological environment of the production place affects the composition and content of TCMs, and the variations in its internal components lead to different quality of TCMs [13]. Bai demonstrated that CR from different producing areas in Gansu has great differences. This study determined the content of metal elements in CR from different producing areas and differentiated CR from different producing areas in Gansu by combining stoichiometric technology [11]. This is a reliable means of identification as well. However, expensive instrumentation was needed for the analysis of the metal content. At the same time, the amounts of elements in CR are little, and they are not the main active ingredients that influence its efficacy. Therefore, it is necessary to establish a less expensive and more convenient discrimination method that can accurately reflect the quality of CR. In addition, kneading processing technology is a traditional processing method of TCM which has an important impact on the quality of TCM. In the original processing, some TCMs such as CR, Polygonati odorati Rhizoma, Notoginseng Radix et Rhizoma, Dendrobii Caulis, Ophiopogonis Radix, etc., need to be kneaded when processing. Beyond that, storage is also a serious factor affecting the quality of TCM. If the storage time is too long or the storage method is not proper, some deterioration phenomena of TCM will occur, such as mold, moth infestation, oil, discoloration, weathering, adhesion, decay and odor loss, which will absolutely affect the quality and clinical efficacy of the TCM [14]. Therefore, a question arises: How to distinguish kneaded and unkneaded CR on the market, or good quality CR and deteriorated CR during storage? This is an urgent problem to be solved. However, the related research is limited, and it is necessary to fill in the blank of this part of research.

**Figure 1:**
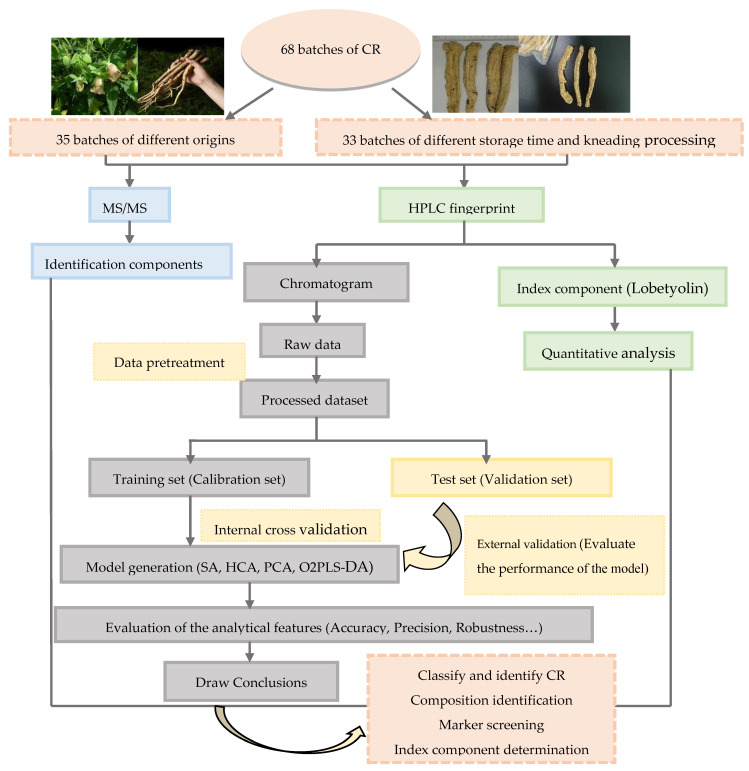
Work flowchart.

In order to ensure the effectiveness and safety of using herbs in therapeutic settings, quality assessment of herbs has received increasing attention in recent years [15,16]. Hence, it is of great importance to distinguish CR of high quality and poor quality. Here, 35 batches of CR from six different origins and 33 batches of CR from different storage time or kneading processing were studied, employing high-performance liquid fingerprint technology and stoichiometric technology. To differentiate CR from six different origins, different storage time and kneading processing, various chemometric techniques such as SA, HCA, and PCA mixed with O2PLS-DA were used. In addition, the characteristic chemical markers of different kinds of CR samples were screened and high-quality CR from different origins, storage time and kneading processing were obtained by analyzing the contents of Lobetyolin and marker components. The identification and quality control (QC) of CR of different origins, storage time and kneading processing were achieved. Finally, some components were identified by tandem mass spectrometry (MS/MS). The results of this study may be a scientific foundation for the authentication of CR. Simultaneously, the results are expected to provide some theoretical basis for the high value utilization of CR. The workflow of this study is shown in Figure 1.

## 2. Results and Discussion

### 2.1. Validation of the Method

Calculating the RSDs of the peak relative retention time (RRT) and relative peak area (RPA) allowed the researchers to assess the method’s accuracy, stability, and repeatability. The precision of the established analytical method was good, as shown by the RSDs of RRT and RPA, which were in the ranges of 0.03–0.89% and 0.42–2.84%, respectively. The suggested approach has strong repeatability, as evidenced by RSDs of RRT and RPA for the repeatability test being 0.02–0.50% and 0.43–2.60%, respectively. Additionally, the stability test’s RSDs for RRT and RPA were less than 0.68 and 2.69%, respectively, indicating that it was possible to evaluate the samples in 24 h. The results show that HPLC is an effective and satisfactory method for fingerprint analysis. 

**Figure 2:**
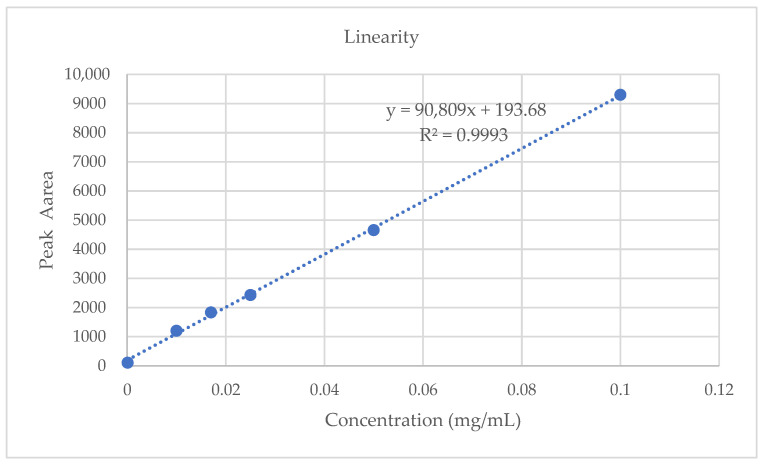
Standard curve of Lobetyolin.

After drawing the standard curve of the average peak area and concentration of Lobetyolin, a linear regression analysis was conducted. It was found that the linear relationship of the analytical component was good (R^2^ > 0.9993 and *p* < 0.05). The regression equation was y = 90,809x + 193.68 and the linear range was 0–0.100 mg/mL, as shown in Figure 2. The limit of detection (LOD) and the limit of quantification (LOQ) measured by the signal to noise ratio (S/N) were 0.031 µg/mL and 0.102 µg/mL, respectively. The recovery rate for Lobetyolin was 100.98% on average. The aforementioned statistics demonstrate the stability and dependability of the experiment’s methodology.

### 2.2. Chromatographic Profiles and Similarity Analysis of Codonopsis Radix

#### 2.2.1. Chromatographic Profiles

**Figure 3:**
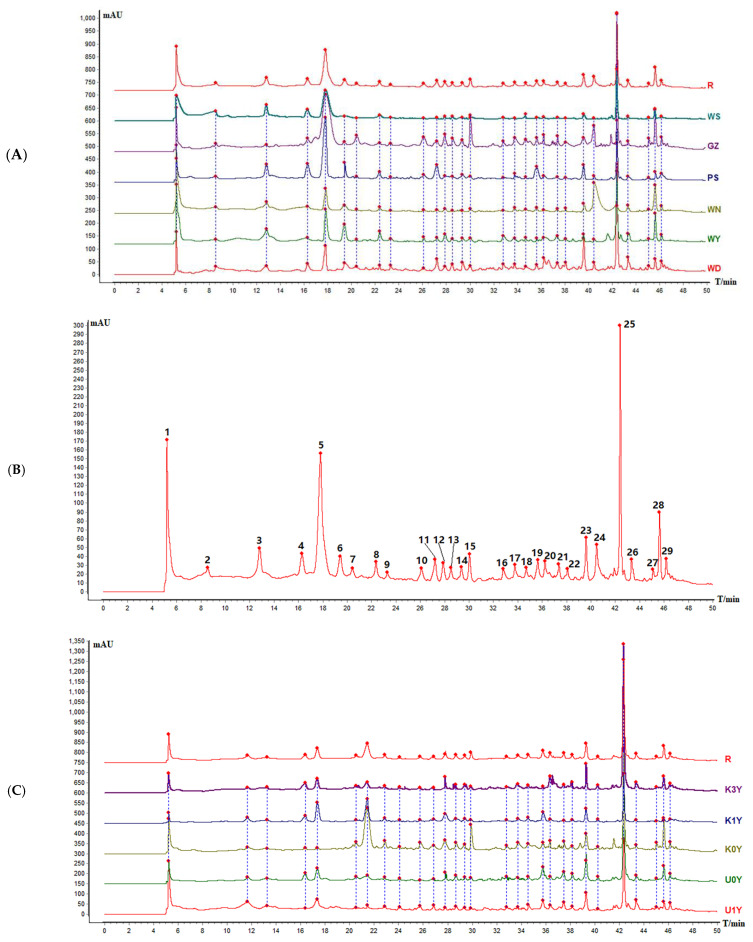

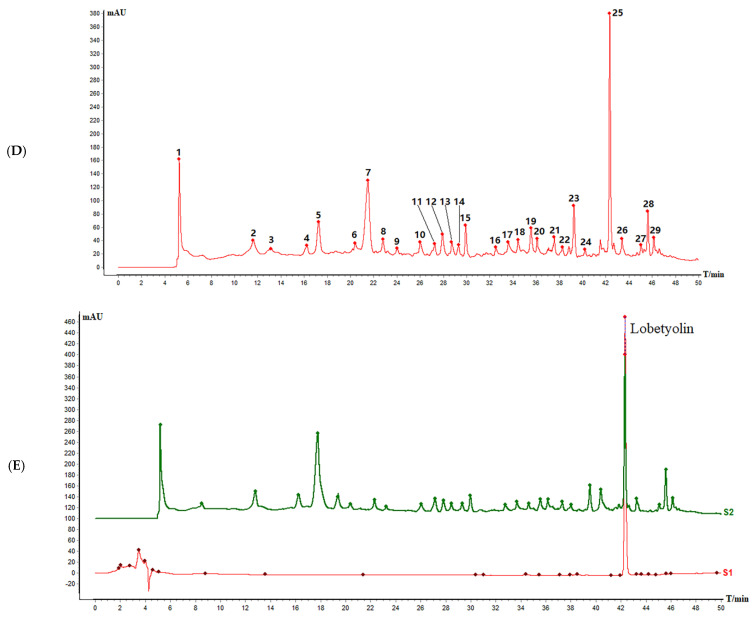
HPLC fingerprint of samples. ((**A**) Reference chromatograms of different origins; WD: Reference chromatograms of Wen, Gansu; WY: Reference chromatograms of Weiyuan, Gansu; WN: Reference chromatograms of Weining, Guizhou; PS: Reference chromatograms of Pingshun, Shanxi; GZ: Reference chromatograms of Ganzi, Sichuan; WS: Reference chromatograms of Wushan, Chongqing. (**B**) Standard fingerprint of different origins; (**C**) Reference chromatograms of different storage time and kneading processing; U0Y: Reference chromatograms of unkneaded, store for 0 years; U1Y: Reference chromatograms of unkneaded, store for 1 year; K0Y: Reference chromatograms of kneaded, store for 0 years; K1Y: Reference chromatograms of kneaded, store for 1 year; K3Y: Reference chromatograms of kneaded, store for 3 years. (**D**) Standard fingerprint of different storage time and kneading processing. (**E**) Chromatogram of content determination of index components, S1: Chromatogram of Lobetyolin reference solution; S2: Chromatogram of sample solution).

For the quality control of the herbs, chromatographic profiles were effectively applied to reveal the chemical information of the botanical products and discriminate the different CR samples. Here, under optimal conditions, the HPLC profiles of the 72 batches of CR from different origins, storage time and kneading processing in China were obtained. The chromatograms of the samples were inputted into the Similarity Evaluation System for Chromatographic Fingerprint of TCMs software (Chinese Pharmacopeia Commission, version 2012.130723) to generate the reference fingerprints of different CRs. The reference fingerprints of WD and K0Y were employed as the reference spectra. The time window width was set to 0.3 min. The mean fingerprint method was employed to generate the standard chromatograms of the different origins, storage time and kneading processing. It was found that a total of 29 peaks were displayed in the chromatographic profiles (Figure 3B,D), and peak No. 25 was Lobetyolin (Figure 3E). Reference chromatograms of different production areas (PS, GZ, WD, WY, WS, WN) were generated (Figure 3A). The reference chromatograms of CR from different storage time and kneading processing (U0Y, U1Y, K0Y, K1Y, K3Y) were generated in the same way (Figure 3C).

It could be concluded, from Figure 3A, that the fingerprints of the different samples varied significantly. Further, the peak area of No. 4 was the largest for PS while WN and WY possessed negligible contents of this ingredient. The peak area of No. 5 was the largest for GZ and PS. The peak area of No. 6 was the largest for WY and PS. The peak area of No. 7 was the largest for GZ while the other origins possessed negligible contents of this ingredient. The peak areas of No. 10–22 were the largest for GZ and WD while WS and WN possessed negligible contents of this ingredient. The peak area of No. 15 was the largest for GZ. The peak areas of No. 11 and No. 19 were the largest for PS. The peak areas of No. 23 and No. 26 were the largest for WD. The peak area of No. 24 was the largest for WN. The peak area of No. 28 was the largest for GZ, WN and WY. At the same time, analysis of the non-common peaks shows that there was an obvious peak at 41.513 min in WY and GZ, but none in others during the same moments. These results indicated that CR varies greatly among different producing areas. By analyzing the sample peak area of the common peaks as shown in Figure 3C, it is not difficult to note that there exists a difference in chemical component content between samples from different storage time and processing. It can be concluded by comparing U0Y and K0Y that the peak areas of No. 1, 7, 8, 10–15, 27 and 28 were greater in K0Y, but smaller in U0Y, indicating that kneaded CR can retain and improve the contents of these components. On the contrary, the peak areas of No. 4, 5, 19 and 23 were greater in U0Y and smaller in K0Y, indicating that the contents of these four components could be reduced by kneading. It can be concluded by comparing U0Y and U1Y that the components of unkneaded CR were significantly reduced after storage for one year. However, it can be seen by comparing U1Y and K1Y that kneading can reduce the reduction in component content in the storage process and can retain the active component. It can be concluded by comparing K0Y, K1Y and K3Y that the content of each component decreases to some extent with the extension of storage time. An interesting phenomenon is that the two components (No.4 and 5) reduced after kneading are gradually regenerated during storage. At the same time, analysis of the non-common peaks shows that there was obvious peak at 41.513 min in K0Y, but none in others during the same moments. These results indicated that the traditional kneading process of CR is of great significance. At the same time, it is suggested that the storage time of CR should not be too long, otherwise it affects the effective ingredients and ultimately affects the clinical efficacy.

These results indicated that there were significant differences in the composition of CR from different origins, storage time and kneading processing. Our findings support the possibility that variables including origin, kneading processing, and storage duration could affect the constituents of the CR. This outcome is also in line with a prior study that compared CRs from Gansu Province using stoichiometric methods [11]. In addition, the reference chromatograms can be used to compare the fingerprint chromatograms of CRs from unknown production areas, storage time and kneading processing and thus to predict the CR with unknown information. However, fingerprint analysis makes it difficult to identify the characteristic peaks that greatly influence the discrepancies. As a result, it is essential to use objective techniques to examine the fingerprint data that has been gathered, such as integrating fingerprint analysis with chemometric techniques.

#### 2.2.2. Similarity Analysis of *Codonopsis* Radix

The Similarity Evaluation System for Chromatographic Fingerprint of TCMs software (Chinese Pharmacopeia Commission, version 2012.130723) was employed to calculate the correlation coefficient by median date. The similarity is a measure of the distance between samples, and a stronger correlation is observed when the value is closer to 1 [17]. Table 1 and Table 2 show the similarity among samples from the same origins, storage time and kneading processing. The similarities were all >0.900, which indicates high intergroup similarity. Table 3 and Table 4 show the similarity among samples from different origins, storage time and kneading processing. The similarities among the samples of different origins were in the range of 0.620–0.954, which indicated that there were significant differences between samples of different origins. The similarities among the samples of different storage time and kneading processing were in the range of 0.700–0.967, which indicated that CR from different storage time and kneading processing had a certain difference.

The aforementioned findings are in line with what we anticipated, which was that the multifactorial parameters considerably changed how similar the various samples were to one another. Our findings concur with those from earlier studies [18,19,20]. It is well-known that the composition and content of chemical components of Chinese herbs change with soil conditions, cultivation measures and climate factors in the growth process [21,22,23,24,25]. In addition, relevant studies have shown that the primary processing, storage time and storage method of TCMs also affect the quality of Chinese herbs [26,27,28,29,30]. This may help to explain why the correlation coefficients for the samples from different origins, storage time and kneading processing in our investigation varied. The SA results showed that sample quality was not evenly distributed. However, SA was only used to determine sample correlation coefficient, and it was unable to classify, identify, or assess sample quality. As a result, it was required to carry out the following analyses.

### 2.3. Multivariate Statistical Analysis

#### 2.3.1. Hierarchical Cluster Analysis

**Figure 4:**
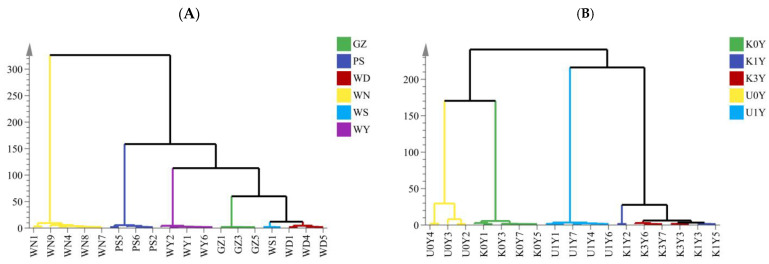
Dendrogram of the hierarchical clustering of CR. ((**A**) Different origin; (**B**) Different storage time and kneading processing).

HCA, a well-known unsupervised pattern recognition approach of data analysis, was used to categorize CRs with different origins, storage time and kneading processing based on the characteristics of chromatographic peaks and to study the consistency between the batches of CRs with the same origins, storage time and kneading processing and the similarities and differences between CRs with different origins, storage time and kneading processing. RPAs of the remaining peaks were estimated using the reference peak of No. 25 (Lobetyolin) because it had a consistent area and good peak shape. To categorize the various samples, the associated RPA data were loaded into the SIMCA 14.1 software. The hierarchical and squared Euclidean distances were used to calculate the cluster dendrogram results. According to their origins, storage time and kneading processing, all of the samples were categorically organized into major clusters (Figure 4). When the classification distance was 20, 35 batches of CR from different origins were divided into five categories. Among them, WN, PS, WY and GZ were clustered into one category, which meant the CRs from WN, PS, WY and GZ samples had good consistency among the same producing areas. The remaining samples (WS and WD) were clustered into another category, indicating that the samples from the two producing areas were highly similar. When the classification distance was 10, CRs from different origins can be divided into six categories according to six different origins as shown in Figure 4A. When the classification distance was 50, 33 batches of CR from different storage time and kneading processing were grouped into four categories (Figure 4B). Among them, U0Y, K0Y and U1Y were clustered into one category, indicating that there is a great difference between U0Y, K0Y and U1Y. The remaining samples (K1Y and K3Y) were clustered into another category, indicating that K1Y and K3Y were highly similar. K1Y and K3Y were consistent with each other in quality.

These findings showed that the metabolites in the samples of different origins, storage time and kneading processing varied significantly. The results of the cluster analysis showed that the metabolite compositions were somewhat influenced by the origins, storage duration, and kneading procedure. The accurate classification of the CR samples was one of the goals of this study. As could be seen, the dendrogram-estimated categorization trend of the samples showed significant discrimination between different samples and decent clustering within the same samples. However, HCA displayed a few drawbacks, including poor visualization and the inability to select out variables that were not related to the categorical variates. Therefore, other multivariate statistical analyses need to be applied in order to further distinguish the samples.

#### 2.3.2. Principal Component Analysis

By analyzing the relationship between the chromatographic peak regions to successfully group samples and identify representative peaks in the chromatogram, PCA was utilized to simplify the complicated data of the CR fingerprints [31,32]. The datasets of RPAs were subjected to PCA using the SIMCA 14.1 software in order to gain an overview of the data structure as well as the distribution, similarities, and differences of the analyzed samples. The peaks were examined to determine whether they could be used to separate CR samples and to ascertain which peaks were the most instructive in doing so. The peak areas of 29 common peaks of CR were used as variables, and the extracted PCs were used to represent the information contained in the original data. The first four PCs delivered the greatest eigenvalues. Therefore, PC1, PC2, PC3 and PC4 were utilized for further analyses. The cumulative contribution rate of the first four PCs reached 73.7% and 79.6% for the PCA models of the different origins, storage time and kneading processing, respectively, indicating the good fitting ability of models. 

**Figure 5:**
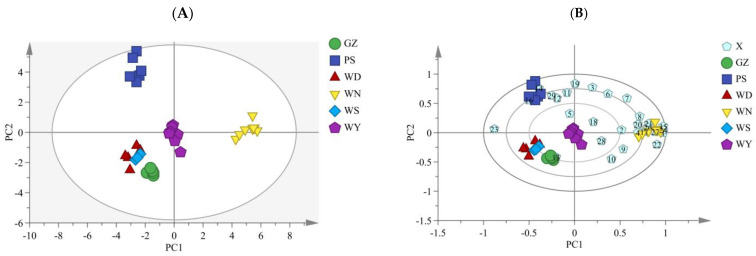

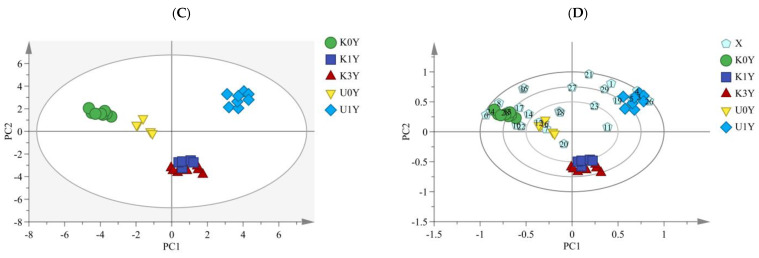
PCA score plot and biplot of CR. ((**A**) PCA score plot of different origin; (**B**) Biplot of different origin; (**C**) PCA score plot of different storage time and kneading processing; (**D**) Biplot of different storage time and kneading processing).

The distribution of the samples was shown on PCs, which also made it possible to analyze how well the samples were discriminated (Figure 5). Based on the PC scores of CR (Figure 5A), 35 batches of CR from different origins were divided into four categories according to the sample source. PS1–PS6 were clustered into one category, WN1–WN9 were grouped together, WY1–WY7 were gathered together, and GZ were adjacent to WD and WS. In addition, PCA results showed that WN samples were different from the other samples distributed on both sides of the longitudinal axis, indicating that the WN samples were distinctly different from the rest. Moreover, according to the PCA biplot (loading and score plot) of different origins (Figure 5B), the content of peaks No.1, 4, 8, 15, 17, 20, 21, 24, 26 and 27 in the WN cultivation area was higher, the content of peaks No.14 and 16 in the PS cultivation area was higher, and the content of peak No.13 was higher in the GZ cultivation area based on the distance between the variable (common peaks) and the sample. These results indicated that these peaks were the most important components for distinguishing CR from different origins, which should be considered when analyzing the differences in origins and the quality of CR. It can be clearly seen from Figure 5C that 33 batches of samples from different storage time and kneading processing were divided into four categories. U1Y1–U1Y8 were clustered into one category, K0Y1–K0Y8 were grouped together, U0Y1–U0Y5 were gathered together, and K1Y and K3Y were assembled together. In addition, PCA results showed that the kneaded samples were far away from the unkneaded samples, indicating that kneading has a great influence on the quality of Chinese herbs. U0Y and U1Y were distributed on both sides of the longitudinal axis, indicating that storage time affects the quality of CR. The distinction between K1Y and K3Y is not obvious, which means that the ingredients in CR can be better preserved after kneading so as to prevent its quality from declining in the storage process. Moreover, according to the PCA biplot (Figure 5D), the content of peaks No.3, 4, 5, 19 and 26 in U1Y was higher, the content of peaks No.7, 12 and 16 in U0Y was higher, the content of peak No.8, 10, 13, 17, 22, 24 and 28 was higher in K0Y, indicating that these peaks were the most important components for distinguishing CR from different storage time and kneading processing.

In line with the findings of SA and HCA, PCA data indicated that there was a significant chemical difference between the samples. Varied assessment techniques reflect the varied qualities of the same data in different ways, as shown by a comparison of SA results with those of HCA and PCA. PCA clearly separated the samples of different origins, storage time and kneading processing, providing a crucial tool for CR identification. It was employed in some relevant research to identify and separate CR from different origins and varieties [11,33,34], but the samples from different kneading processing and storage times were limitedly included. The results of our study, combined with those of earlier studies, can offer a thorough method for the identification and quality assessment of CR from various sources.

#### 2.3.3. Two-Way Orthogonal Partial Least Square with Discriminant Analysis

**Figure 6:**
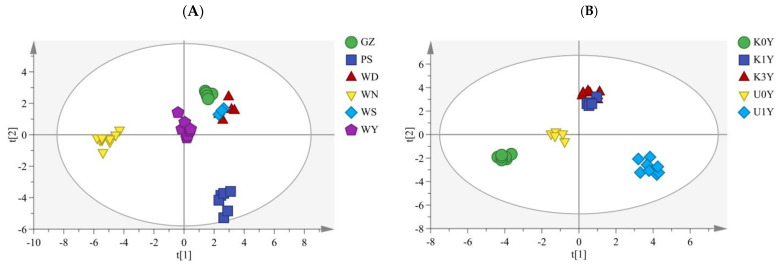
O2PLS–DA score plot of CR. ((**A**) Different origin; (**B**) Different storage time and kneading processing).

**Figure 7:**
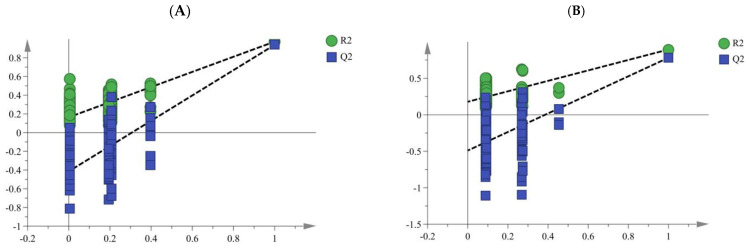
Permutation test of the O2PLS–DA model. ((**A**) Different origin; (**B**) Different storage time and kneading processing).

Two-way orthogonal partial least square (O2PLS) is a multivariate projection method that, by removing the so-called structured noise from two data blocks X and Y, extracts linear correlations. By providing the appropriate dummy variables, O2PLS can also be used to perform discriminant analysis (DA). The key advantages of the two-way orthogonal partial least square with discriminant analysis (O2PLS–DA) technique are the simplification of the model and the clarification of the distinctions between the various samples. This technique can be used to differentiate between different data classes, improve class separation, make interpretation simpler, and find possible biomarkers [35,36,37]. Here, the O2PLS–DA models were established with the score plot, as shown in Figure 6, displaying a very good discrimination between samples. The determination coefficient (R2X), response variance ratio (R2Y) and predictive ability (Q2) of the O2PLS–DA model from different origins were 0.778, 0.927 and 0.888, respectively (Figure 6A). R2X, R2Y, and Q2 of the model from different storage time and kneading processing were 0.822, 0.923 and 0.862, respectively (Figure 6B). These numbers demonstrate the O2PLS–DA model’s stability and dependability [38]. The majority of the samples could be distinguished into distinct groups according to the different origins, storage time and kneading processing, as shown in Figure 6. Among them, WS and WD samples were similar and could not be differentiated. In addition, due to the high similarity between K1Y and K3Y, the O2PLS–DA model cannot distinguish them well. In general, O2PLS–DA performed better and was a better fit for the discrimination than PCA and HCA. In order to assess the validity of the established O2PLS–DA models, 200 random permutation tests were conducted. The regression line of the permuted ones intersected the vertical axis, and the R2 and Q2 values were greater in the original O2PLS–DA models than in the permuted ones (Figure 7). These findings indicate the established O2PLS–DA model’s high goodness of fit as well as predictability.

The O2PLS–DA models were created in this case, and the score plots showed that the samples could be distinguished clearly, supporting the findings of the SA, HCA, and PCA analyses. O2PLS–DA performed better and was more accurate than PCA and HCA for discriminating because it could filter out data variations that were unrelated to the independent and categorical factors. We used a really straightforward strategy to correctly categorize and identify CR in our research from various sources. Unfortunately, due to restrictions on sampling, a relatively small number of samples were employed in this study. This obstacle, however, had little impact on our conclusion. The results showed that the sample classification was clear and would have provided the same outcomes even if we had increased the number of samples.

**Figure 8:**
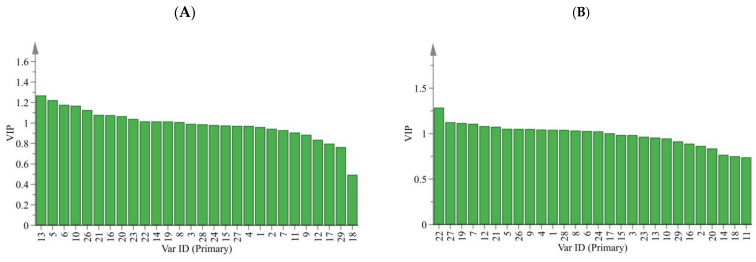
VIP predicative plot of each variable (confidence intervals at 95% level). ((**A**) Different origin; (**B**) Different storage time and kneading processing).

Moreover, the variable influence on projection values (VIP > 1) were chosen as differential marker candidates for each CR. A variable’s importance in explaining the variance in the dataset and the relationships across groups is shown by its VIP predictive value [39]. Finally, 13 marker compounds, consisting of peaks 13, 5, 6, 10, 26, 21, 16, 20, 23, 22, 14, 19 and 8, were obtained to identify the different origins (Figure 8A). Further, 16 marker compounds, consisting of peaks 22, 27, 19, 7, 12, 21, 5, 26, 9, 4, 1, 28, 8, 6, 24 and 17, were obtained to identify the different storage time and kneading processing (Figure 8B). The O2PLS–DA model could clearly and systematically identify six CR origins and five CR of different storage time and kneading processing.

### 2.4. Determination of Lobetyolin in Codonopsis Radix Samples

Lobetyolin is one of the most important active components of CR which has good anticancer effects [40,41,42], exerts cytotoxic protection [43] and was also used to treat chronic kidney disease [44]. In the Chinese Pharmacopoeia (2020 edition), Lobetyolin was also employed as a distinguishing component to identify CR via thin-layer chromatography (TLC). Here, 68 batches of CR samples were taken and prepared into sample solutions according to the method described in “item 3.3.”. After the sample solutions were injected into the HPLC and analyzed under the chromatographic conditions in “item 3.4.”, the peak area was recorded, and the contents of Lobetyolin were calculated according to the regression equation (Figure 2). It can be seen that the contents of Lobetyolin in CR samples from different regions were quite different (Table 5). The results showed that the contents of the WD area were higher, followed by GZ and WY, and the lowest were PS, WN and WS. It is very interesting that the WD, GZ and WS samples were very similar and could not be distinguished in the previous analysis. However, the content of Lobetyolin in the samples from the three producing areas was quite different, especially in WS samples. This means that samples from these three regions can be distinguished by the content of the index component Lobetyolin. Table 6 illustrates the variation in the content of Lobetyolin in CR samples from different storage time and kneading processing. It is obvious that the content of Lobetyolin decreased with the extension of storage time, and kneading processing can prevent the content of Lobetyolin from decreasing. Therefore, it is recommended to knead CR in the initial processing while reducing its storage time. 

**Figure 9:**
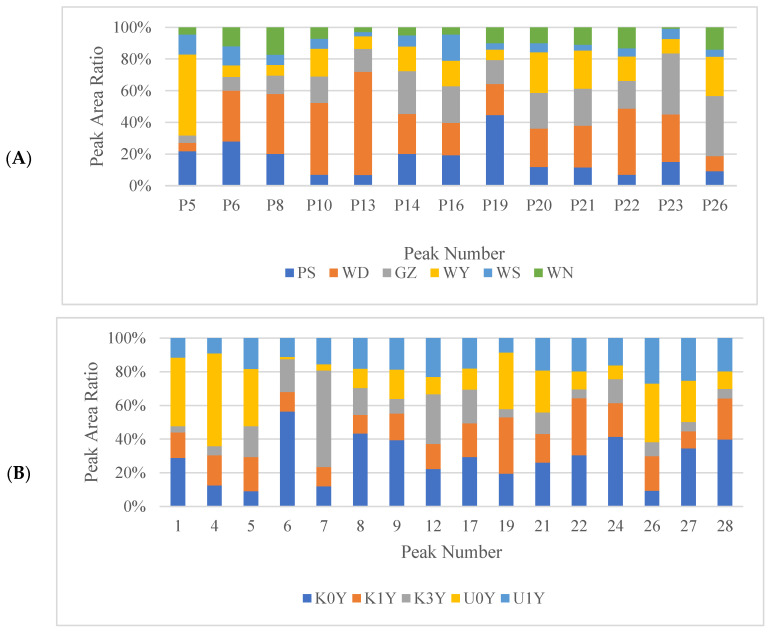
The distribution of marker compounds. ((**A**) Different origin; (**B**) Different storage time and kneading processing).

As is known, the content of an active ingredient cannot represent the overall quality of a sample. Moreover, a single active substance is not responsible for the overall pharmacological potency. Therefore, in order to comprehensively evaluate the quality of CR from different sources, we simultaneously analyzed the peak area changes in several different metabolites as quantitative expressions. Figure 9A reveals the distribution of each peak in CR from different origins. Comprehensively, the contents were significantly high in the WD samples, followed by PS, WY and GZ, and the content was the lowest in WS and WN. This indicated that the quality of CR in Gansu and Shanxi Province was the best, followed by that in Sichuan Province, while that in Guizhou Province was relatively poor. These results demonstrated a large difference in the accumulations of 13 marker compounds (peaks 13, 5, 6, 10, 26, 21, 16, 20, 23, 22, 14, 19, 8) in different origins. We also analyzed the 16 marker compounds (peaks 22, 27, 19, 7, 12, 21, 5, 26, 9, 4, 1, 28, 8, 6, 24, 17) in CR from different storage time and kneading processing (Figure 9B). The contents of the 16 marker compounds were extremely high in K0Y samples, indicating that CR was of good quality after kneading. Simultaneously, by comparing the peak area of 16 marker compounds of CR with different storage times, it was obvious that the longer the storage time, the worse the quality of CR. The best-quality CR is a fresh one, that is, CR stored for 0 years. Therefore, we could assess the quality of CR by analyzing the characteristic peaks of the fingerprint.

### 2.5. Identification of Chemical Components in Codonopsis Radix Sample

To obtain the chemical information of CR, compounds were identified by the method of UPLC-MS/MS. The negative and positive ion modes were both adopted for MS analysis. Through this analysis, a total of 71 components in the sample were identified by comparing the molecular ion mass and fragment ions with previous data and the reference substances, including twelve aliphatic acyl and derivatives, eight alkaloid and derivatives, eight flavonoids and derivatives, five organic acids and derivatives, eight phenols and derivatives, seven phenylpropanoids and derivatives, six terpenoids and derivatives, five amino acid and derivatives, six glycoside and six miscellaneous. These components are responsible for the differences between samples. Detailed data are shown in Table S1 of the Supplementary Files.

## 3. Materials and Methods

### 3.1. Chemicals and Materials

Lobetyolin standards were purchased from the National Institutes for Food and Drug Control (Beijing, China). Acetonitrile (HPLC-grade and MS-grade) was purchased from Fisher Scientific USA (Waltham, MA, USA). Ethanol was purchased from Tianjin Damao Chemical Reagent Factory (Tianjin, China). Methanol (MS-grade) was purchased from CNW Technologies GmbH (Dusseldorf, Germany). Formic acid (MS-grade) was purchased from Sigma Chemical Co. (St. Louis, MO, USA). Purified water was obtained from Wahaha Group Co., Ltd. (Hangzhou, China). Different batches of CR were collected from different provinces of China. All CR samples were authenticated as the dried roots of *Codonopsis pilosula* (Franch.) Nannf. (*C. pilosula*), *Codonopsis pilosula* Nannf. var. modesta (Nannf.) L.T.Shen (*C. pilosula* var. modesta), and *Codonopsis tangshen* Oliv. (*C. tangshen*) by Prof. Fude Yang from the Department of Traditional Chinese Medicine authentication at Gansu University of Traditional Chinese Medicine. The details of samples are shown in Table 7. CR samples were deposited at the department of TCM identification in Gansu University of Traditional Chinese Medicine.

### 3.2. Instrumentation

A Waters ACQUITY Arc (U)HPLC system (Milford, MA, USA) equipped with an automatic sample manager (FTN—R), a quaternary solvent manager—R, a Waters 2998 photodiode array (PDA) detector and a Waters empower software (version 7.0.3471.914) workstation was used. MS analysis was performed by a Thermo Fisher Orbitrap Exploris 120 mass spectrometer (Waltham, MA, USA). The extraction of the sample was sonicated using a DT1028H sonicator (Bandelin electronic GmbH & Co. KG, Berlin, Germany). For processing of the raw samples, a TDL-5—A centrifuge (Shanghai Anheng Scientific Instrument Factory, Shanghai, China) and an XQ100 grinder (Shanghai Guangsha Industry and Trade Co., Ltd., Shanghai, China) were employed. Samples were dried using a DHG—9140A oven from Shanghai Jinghong Experimental Equipment Co., Ltd. in China. A KERN ABT100—5M analytical balance (KERN and SOHN GmbH, Balingen, Germany) was used to weigh the samples.

### 3.3. Preparation of Codonopsis Radix Extracts

Ultrasonic preparation was used to create CR extracts. In a nutshell, the samples were dried in an oven at 50 °C for 4 h. Then, the samples were put in a desiccator to cool to ambient temperature (22–25 °C). The materials were then run through an 80-mesh filter after being ground with a grinder. A piece of the powder weighing 1.0 g was placed in a 100 mL Erlenmeyer flask along with 15 times as much 50% ethanol (*w*/*v*). The mixture was extracted using an ultrasonic device at 50 °C for 30 min. The solutions were centrifuged at 10,000 rpm for 10 min, and then filtered for HPLC analysis through a 0.45 m membrane filter. 

### 3.4. Chromatographic and Mass Spectrometry Conditions

Chromatographic separation was performed at ambient temperature (22–25 °C) employing a Sepherisorb ODS2 C18 column (5 μm, 4.6 mm × 250 nm). The optimal mobile phase was composed of water (A) and acetonitrile (B) under a gradient elution program at a flow rate of 0.8 mL/min: 0–2 min, 2% B; 2–6 min, 2–4% B; 6–15 min, 4–10% B; 15–25 min, 10–15%B; 25–35 min, 15–25% B; 35–40 min, 25–35% B; 40–50 min, 35–40% B. The injection volume and detection wavelength were 20 µL and 210 nm, respectively. 

Compounds in the real samples were identified using MS/MS spectra. The investigation of positive and negative ions using electrospray MS/MS was conducted. The following relevant parameters were optimized and set: Ion Transfer Tube Temperature: 350 °C; Vaporizer Temperature: 350 °C; Sheath Gas Flow Rate: 35 Arb; Aux Gas Flow Rate: 15 Arb; Full MS Resolution: 60,000; MS/MS Resolution: 15,000; Energy of collision, 16/32/48 in NCE mode; 5.5 kV (positive) and −4 kV (negative) for spray voltage.

### 3.5. Method Validation

#### 3.5.1. Validation of the HPLC Fingerprint Method 

##### Precision

According to the chromatographic requirements, one sample extract (WD1) was constantly injected (five times in a single day). Calculations were performed for the relative retention time (RRT) and relative peak area (RPA). The relative standard deviation (RSD) of the precision, as determined by the marker compounds in the sample, was used to express it.

##### Stability

One of the sample extracts (WD1) that was held at 20–25 °C and reanalyzed after 0, 2, 4, 6, 8 and 24 h of storage was used to test the sample stability. As a stability indicator, the RSDs of RRT and RPA were acquired.

##### Repeatability

Five repetitions of the extraction process were used to assess its repeatability and that of the analysis. After injecting the samples, the chromatograms were captured in accordance with the chromatographic conditions, and the RSDs of RRT and RPA were acquired.

#### 3.5.2. Validation of the Quantitative Method

A CR reference solution (Lobetyolin) with a concentration of 1 mg/mL was prepared with a 50% ethanol solution. It was then diluted into different concentrations with a 50% ethanol. After that, the HPLC was used to examine the standard solutions at various concentrations using the chromatographic method. The concentration (mg/mL) was used as the abscissa (X) and the mean peak area (Y) as the ordinate for plotting the calibration curves. The concentration of a standard solution with an S/N of 3 was the limit of detection (LOD) and the concentration of the standard solution with an S/N of 10 was the limit of quantification (LOQ), respectively.

The accuracy of the index Lobetyolin was assessed using the recovery test. From the same batch of CR powder (WD1, 1.000 g), six aliquots were taken and precisely weighed. The correct amounts of Lobetyolin were then added to this. After that, the solution was made as previously described, and chromatographic analysis was carried out. Three tests were performed on each sample. Calculating the ratio of the detected amount to the added amount allowed for the evaluation of the average recovery percentage.

### 3.6. Data Collection and Chemometric Methods

The chromatographic fingerprint of CR was created using the Similarity evaluation system for chromatographic fingerprint of TCM software (China Committee of Pharmacopeia, 2012 version), which also generated the simulative mean chromatogram and the characteristic peaks. The correlation coefficients of the entire chromatographic patterns among all of the samples were calculated. Each characteristic peak’s RPA and RRT were determined in comparison to the internal reference peak. Furthermore, chemometric analyses were carried out using RPA of each distinctive peak. Chemometric analyses such as HCA, PCA, and O2PLS-DA were used based on the soft independent modeling by class analogy (SIMCA) program (v. 14.1, 2015, Umetrics, Umea, Sweden; www.umetrics.com, accessed on 16 March 2023).

## 4. Conclusions

In view of the differences and the complexity of components of CR from different sources, this study established a quality evaluation method and discrimination model of CR via an approach combining chemometric methods and fingerprint analysis, which was successfully applied to evaluate and identify CR of different origins, storage time and kneading processing. Reference chromatograms of different CR were established to provide a reference for the quality evaluation and prediction of different CR. The fingerprint analysis and the SA, HCA, PCA, and O2PLS–DA results showed that there are certain differences in the chemical constituents between these CR samples. In particular, the O2PLS–DA model can well distinguish and identify CR of different origins, storage time and kneading processing. Moreover, 13 and 16 major compounds were identified as marker compounds for the discrimination of CR from different origins, storage time and kneading processing, respectively. These compounds can be used to guide the quality control of CR. By measuring the relative content of the marker components and the exact content of Lobetyolin, the results showed that the contents of these components vary greatly among different CR samples. In general, the quality of CR from Gansu was the best, followed by Shanxi and Sichuan. The quality of CR from Chongqing and Guizhou was poor. At the same time, the quality of CR was the best when it was kneaded and stored for 0 years. Finally, a total of 71 components of CR were identified by UPLC-MS/MS, which were responsible for the differences between samples. In this study, there were significant differences between samples from different sources. Variations in the quality and composition of CR based on the variety, sources, harvest time, growing year, storage period, and processing of CR are the cause of the poor consistency across batches [45,46]. To solve the problem of inconsistent quality, we can regulate and control the planting, harvesting, processing and storage of CR. We can also control the environmental factors to ensure the consistent quality of CR.

Quality control of TCM is rather challenging because of its complex composition as well as the fact that a single component is not responsible for the overall pharmacological potency. Fortunately, chemometric methods could evaluate TCMs comprehensively. Various results complemented and verified each other, thus ensuring the accurate classification and identification of TCMs. Accordingly, we also established a comparative method of discriminating and evaluating the quality of CR from different origins, storage time and kneading processing. These methods will help prevent possible side effects and poor quality resulting from incorrect identifications. Additionally, the technique used here served as a guide for the development and utilization of CR as well as for the assessment of the authenticity, quality, and development of TCMs or other related food and medications. However, the quality of TCMs is ultimately reflected in its clinical efficacy. Therefore, it is necessary to validate this method by combining it with more comprehensive pharmacological comparisons of CR from different sources in the future.

## Figures and Tables

**Table 1:** Similarity of samples from same origins.

Sample	Similarity
PS	0.921–0.998
GZ	0.960–0.997
WD	0.993–0.999
WY	0.989–0.998
WS	0.940–0.994
WN	0.906–0.998

**Table 2:** Similarity of samples from same storage time and kneading processing.

Sample	Similarity
U0Y	0.938–0.998
U1Y	0.939–0.994
K0Y	0.953–0.997
K1Y	0.925–0.996
K3Y	0.957–0.998

**Table 3:** Similarity of samples from different origins.

	WD	WY	WN	PS	GZ	WS
WD	1.000	0.787	0.620	0.699	0.856	0.713
WY	0.787	1.000	0.947	0.930	0.880	0.954
WN	0.620	0.947	1.000	0.871	0.785	0.892
PS	0.699	0.930	0.871	1.000	0.859	0.926
GZ	0.856	0.880	0.785	0.859	1.000	0.794
WS	0.713	0.954	0.892	0.926	0.794	1.000

**Table 4:** Similarity of samples from different storage time and kneading processing.

	U1Y	U0Y	K0Y	K1Y	K3Y
U1Y	1.000	0.835	0.700	0.748	0.753
U0Y	0.835	1.000	0.838	0.859	0.967
K0Y	0.700	0.838	1.000	0.858	0.841
K1Y	0.748	0.859	0.858	1.000	0.856
K3Y	0.753	0.967	0.841	0.856	1.000

**Table 5:** Contents of Lobetyolin in CR samples from different origins.

Sample	The Content of Lobetyolin (mg/g)	Number of Samples (n)
PS	0.266	6
GZ	0.578	5
WD	0.625	5
WY	0.314	7
WS	0.236	3
WN	0.247	9

**Table 6:** Contents of Lobetyolin in CR samples from different storage time and kneading processing.

Sample	The Content of Lobetyolin (mg/g)	Number of Samples (n)
U0Y	0.834	5
U1Y	0.430	8
K0Y	0.952	8
K1Y	0.598	5
K3Y	0.276	7

**Table 7:** The information on samples.

	Sample Information	Sample No.
Different origins	Pingshun County, Shanxi Province	PS (PS1–PS6)
	Ganzi County, Sichuan Province	GZ (GZ1–GZ5)
	Wen County, Gansu Province	WD (WD1–WD5)
	Weiyuan County, Gansu Province	WY (WY1–WY7)
	Wushan County, Chongqing Municipality	WS (WS1–WS3)
	Weining County, Guizhou Province	WN (WN1–WN9)
Different storage time and kneading processing	Unkneaded, Store for 0 years	U0Y (U0Y1–U0Y5)
	Unkneaded, Store for 1 year	U1Y (U1Y1–U1Y8)
	Kneaded, Store for 0 years	K0Y (K0Y1–K0Y8)
	Kneaded, Store for 1 year	K1Y (K1Y1–K1Y5)
	Kneaded, Store for 3 years	K3Y (K3Y1–K3Y7)

## Data Availability

Data are contained within the article. In additional, the data presented in this study are available on request from the corresponding author.

## Supplementary Materials

The following supporting information can be downloaded at: https://www.mdpi.com/article/10.3390/molecules28135127/s1, Table S1: Mass spectrometric identification results.

## Sample Availability

Not available.

## Abbreviations

CR: *Codonopsis Radix*; HPLC: high-performance liquid chromatography; O2PLS—DA: orthogonal two-part least squares-discriminant analysis; PCA: principal component analysis; HCA: hierarchical cluster analysis; RSD: relative standard deviation; LOQ: limit of quantification; LOD: limit of detection; TCM: traditional Chinese medicine; PS: Pingshun County, Shanxi Province; GZ: Ganzi County, Sichuan Province; WD: Wen County, Gansu Province; WY: Weiyuan County, Gansu Province; WS: Wushan County, Chongqing Municipality; WN: Weining County, Guizhou Province; U0Y: Unkneaded, Store for 0 years; U1Y: Unkneaded, Store for 1 year; K0Y: Kneaded, Store for 0 years; K1Y: Kneaded, Store for 1 year; K3Y: Kneaded, Store for 3 years.
