# Supplementary material for: Quality Assessment and Classification of Codonopsis Radix Based on Fingerprints and Chemometrics
Source: Molecules. 2023 Jun 29;28(13):5127. doi: 10.3390/molecules28135127 (PMC10343490; doi:10.3390/molecules28135127)
Supplement: Supplementary file 1 [file molecules-28-05127-s001.zip › molecules-2448387-supplementary.pdf]

molecules MDPI

Table S1 Mass spectrometric identification results

No.	t_R /min	Formula	Mass (m/z)	Type	MS^2/(m/z)	Error /ppm	Name	Class
1	7.43	$C_{17}H_{34}O_2$	315.2544	$[M+HCOO]^-$	315.2523;316.2565;297.2446;241.1816;313.2382	1.38	Methyl hexadecanoate	Aliphatic acyl and derivatives
2	12.62	$C_{18}H_{36}O_2$	283.2636	$[M-H]^-$	283.2618;284.2661;79.9573;265.1784;288.8614	1.42	Palmitic Acid ethyl ester	Aliphatic acyl and derivatives
3	8.74	$C_{18}H_{30}O_2$	279.2323	$[M+H]^+$	81.0702;280.2325;95.0859;67.0545;96.0890	1.07	Alpha-Linolenic acid	Aliphatic acyl and derivatives
4	7.65	$C_9H_{16}O_4$	187.0977	$[M-H]^-$	125.0970;187.0984;97.0654;123.0822;57.0345	1.48	Azelaic acid	Aliphatic acyl and derivatives
5	11.42	$C_{24}H_{36}O_4$	389.2680	$[M+H]^+$	389.2632;390.2710;249.1088;95.0858;81.0707	2.66	Cavipetin C	Aliphatic acyl and derivatives
6	9.85	$C_{18}H_{34}O_3$	297.2437	$[M-H]^-$	297.2447;298.2443;171.1034;279.2354;152.9965	2.39	Ricinoleic acid	Aliphatic acyl and derivatives
7	11.82	$C_{18}H_{30}O_2$	277.2174	$[M-H]^-$	277.2183;278.2184;233.1887;59.0136;259.2098	1.32	A-Linolenic acid	Aliphatic acyl and derivatives
8	11.18	$C_{17}H_{34}O_2$	269.2485	$[M-H]^-$	269.2480;270.2533;73.0293;239.2384;71.0136	1.69	Heptadecanoic acid	Aliphatic acyl and derivatives
9	12.51	$C_{18}H_{32}O_2$	279.2331	$[M-H]^-$	279.2353;280.2355;261.2207;59.0141;71.9248	0.31	Linoelaidic acid	Aliphatic acyl and derivatives
10	11.83	$C_{18}H_{34}O_2$	281.2481	$[M-H]^-$	281.2464;282.2515;87.0086;237.1506;57.0345	0.23	Oleic acid	Aliphatic acyl and derivatives
11	0.03	$C_{16}H_{32}O_2$	257.2468	$[M+H]^+$	57.0698;71.0856;257.2491;69.0700;85.1006	0.67	Palmitic acid	Aliphatic acyl and derivatives
12	0.41	$C_{18}H_{36}O_2$	285.2788	$[M+H]^+$	285.2762;71.0856;57.0698;303.0603;55.0543	0.75	Stearic acid	Aliphatic acyl and derivatives
13	3.07	C_9H_9N	130.0662	$[M-H]^-$	130.0667;131.0702;146.0618;87.0447;128.0512	1.26	3-Methylindole	alkaloid and derivatives
14	2.22	C_9H_7NO	144.0457	$[M-H]^-$	144.0457;145.0494;101.0607;71.0143;116.0504	2.26	Indole-3-carboxaldehyde	alkaloid and derivatives
15	0.62	$C_5H_5N_5$	136.0616	$[M+H]^+$	136.0622;137.0593;119.0356;91.0542;94.0405	2.62	Adenine	alkaloid and derivatives

16	2.22	C_8H_7N	118.0653	[M+H]$^+$	118.0654;119.0854;91.0541;105.0444;95.0492	2.36	Indole	alkaloid and derivatives
17	0.56	$C_6H_6N_2O$	123.0552	[M+H]$^+$	123.0547;124.0390;80.0493;96.0445;106.0285	1.57	Nicotinamide	alkaloid and derivatives
18	0.92	$C_6H_{15}N$	102.1278	[M+H]$^+$	102.1271;43.0541;85.1015;57.0698;41.0387	1.71	Hexylamine	alkaloid and derivatives
19	6.90	$C_{16}H_{15}NO_2$	254.1173	[M+H]$^+$	254.1173;212.1071;95.0725;236.1080;159.0441	2.58	Ethyl 3-methyl-9H-carbazole-9-carboxylate	alkaloid and derivatives
20	0.55	$C_5H_{14}NO^+$	104.1070	[M+H]$^+$	104.1071;60.0807;45.0336;87.0439;58.0648	0.03	Choline chloride	alkaloid and derivatives
21	7.46	$C_{15}H_{10}O_5$	269.0456	[M-H]$^-$	269.0465;225.0540;270.0508;241.0522;62.9645	1.56	Galangin	flavonoids and derivatives
22	3.36	$C_{15}H_{10}O_6$	287.0557	[M+H]$^+$	287.0570;153.0175;288.0582;135.0445;227.1085	2.54	Luteolin	flavonoids and derivatives
23	2.05	$C_{22}H_{22}O_{11}$	463.1247	[M+H]$^+$	301.0685;286.0493;463.2744;85.0280;258.0545	1.56	Diosmetin-7-O-beta-D-glucopyranoside	flavonoids and derivatives
24	3.78	$C_{16}H_{12}O_4$	267.0669	[M-H]$^-$	267.0655;252.0417;268.0694;253.0485;223.0405	3.22	Formononetin	flavonoids and derivatives
25	1.13	$C_{15}H_{10}O_6$	287.0557	[M+H]$^+$	287.0571;153.0176;288.0580;239.0803;269.0914	2.31	Kaempferol	flavonoids and derivatives
26	3.13	$C_{22}H_{22}O_9$	431.1349	[M+H]$^+$	269.0807;254.0593;213.0896;270.0858;237.0558	2.11	Ononin	flavonoids and derivatives
27	2.07	$C_{28}H_{32}O_{16}$	625.1778	[M+H]$^+$	317.0644;85.0280;71.0489;302.0427;301.0683	1.31	Isorhamnetin-3-O-nehesperidine	flavonoids and derivatives
28	1.67	$C_9H_{10}O_4$	183.0653	[M+H]$^+$	123.0448;95.0490;155.0696;140.0464;183.0635	1.43	Syringaldehyde	flavonoids and derivatives
29	9.66	$C_{18}H_{30}O_2$	277.2174	[M-H]$^-$	277.2183;278.2186;59.0136;233.2273;64.8628	1.32	Gamma-Linolenic acid	Organic acids and derivatives
30	0.60	$C_4H_6O_4$	117.0194	[M-H]$^-$	73.0292;117.0200;99.0092;59.0141;71.0502	3.65	Succinate	Organic acids and derivatives
31	0.55	$C_4H_6O_5$	133.0143	[M-H]$^-$	116.0070;134.0176;72.0171;115.0039;71.0136	1.93	L-Malic acid	Organic acids and derivatives
32	0.40	$C_8H_4O_3$	149.0229	[M+H]$^+$	149.0226;93.0702;121.0282;67.0288;107.0848	0.35	Phthalic anhydride	Organic acids and derivatives

33	10.83	$C_{21}H_{36}O_4$	375.2501	$[M+Na]^+$	375.2496;376.2555;69.0700;249.1101;81.0702	2.49	Glyceryl linolenate	Organic acids and derivatives
34	2.10	$C_7H_6O_2$	121.0295	$[M-H]^-$	121.0294;122.0332;93.0345;120.0222;92.0266	4.29	P-Hydroxybenzaldehyde	Phenols and derivatives
35	0.60	$C_8H_8O_4$	169.0495	$[M+H]^+$	169.0875;95.0859;81.0700;123.0809;71.0489	2.96	4-Methoxysalicylic acid	Phenols and derivatives
36	1.28	$C_7H_6O_2$	121.0295	$[M-H]^-$	121.0294;122.0335;120.0222;92.0263;93.0346	4.03	3-Hydroxybenzaldehyde	Phenols and derivatives
37	1.10	$C_8H_8O_2$	137.0598	$[M+H]^+$	137.0605;138.0538;122.0368;109.0652;81.0703	1.71	3-Methoxybenzaldehyde	Phenols and derivatives
38	1.17	$C_9H_{10}O_2$	151.0753	$[M+H]^+$	135.0446;152.0696;109.0652;153.0538;107.0490	1.97	3,4-Dimethylbenzoic acid	Phenols and derivatives
39	0.96	$C_8H_{10}O$	123.0804	$[M+H]^+$	123.0547;124.0391;80.0493;95.0491;81.0335	3.27	4-Ethylphenol	Phenols and derivatives
40	0.59	$C_7H_6O_5$	169.0141	$[M-H]^-$	125.0248;169.0133;81.0343;97.0298;69.0347	0.48	Gallic acid	Phenols and derivatives
41	5.12	$C_6H_6O_3$	125.0244	$[M-H]^-$	125.0248;81.0343;126.0278;97.0298;94.9012	3.13	1,2,3-Trihydroxybenzene	Phenols and derivatives
42	1.74	$C_{10}H_{10}O_4$	193.0503	$[M-H]^-$	134.0380;178.0277;193.0503;149.0613;121.0293	1.76	Trans-4-Hydroxy-3-methoxycinnamate	Phenylpropanoids and derivatives
43	0.44	$C_{10}H_8O_5$	209.0446	$[M+H]^+$	209.0434;96.9956;163.1134;135.0805;107.0864	2.68	Fraxetin	Phenylpropanoids and derivatives
44	5.13	$C_{22}H_{26}O_8$	417.1562	$[M-H]^-$	417.1473;138.0319;105.0193;166.0270;267.0699	0.37	Syringaresinol	Phenylpropanoids and derivatives
45	7.58	$C_{11}H_{12}O_3$	193.0860	$[M+H]^+$	193.0870;105.0703;133.0657;165.0922;137.0607	0.20	Myristicin	Phenylpropanoids and derivatives
46	10.38	$C_{29}H_{42}O_{18}$	677.2135	$[M-H]^-$	497.1670;453.1773	0.86	Tangshenoside Ⅰ	Phenylpropanoids and derivatives
47	4.27	$C_{15}H_{14}O_5$	275.0913	$[M+H]^+$	161.0595;275.1982;105.0701;201.0525;133.0656	2.37	Alpha-Pyrufuran	Phenylpropanoids and derivatives
48	1.93	$C_{16}H_{19}NO_2$	258.1488	$[M+H]^+$	258.1480;158.0971;122.0595;216.1395;230.1545	0.95	Coumaperine	Phenylpropanoids and derivatives
49	6.21	$C_{15}H_{20}O_3$	247.1337	$[M-H]^-$	247.1325;203.1428;83.0503;248.1385;187.1137	1.05	Atractylenolide III	Terpenoids and derivatives
50	5.09	$C_{15}H_{18}O_2$	231.1384	$[M+H]^+$	231.1391;185.1333;163.0759;157.1018;213.1270	1.88	Atractylenolide I	Terpenoids and derivatives
51	8.10	$C_{15}H_{20}O_2$	233.1538	$[M+H]^+$	233.1521;187.1487;151.0748;215.1435;177.0910	0.73	Atractylenolide II	Terpenoids and derivatives

52	13.09	$C_{30}H_{48}O_3$	455.3527	$[M-H]^-$	455.3569;456.3623;50.5955;321.2070;71.0501	0.61	Betulinic acid	Terpenoids and derivatives
53	9.06	$C_{15}H_{22}O_2$	233.1547	$[M-H]^-$	233.1545;234.1579;126.9502;218.1280;217.1220	3.20	Confertifolin	Terpenoids and derivatives
54	8.55	$C_{15}H_{24}O_2$	235.1704	$[M-H]^-$	235.1723;236.1750;59.0135;101.9413;220.0397	1.58	Dihydroartemisinic acid	Terpenoids and derivatives
55	0.56	$C_5H_7NO_3$	128.0353	$[M-H]^-$	128.0356;85.0297;129.0191;101.0243;84.0453	2.26	5-Oxoproline	Amino acid and derivatives
56	1.53	$C_9H_{11}NO_2$	164.0717	$[M-H]^-$	147.0456;164.0723;121.0295;72.0090;120.0538	2.00	DL-Phenylalanine	Amino acid and derivatives
57	0.55	$C_5H_9NO_2$	116.0705	$[M+H]^+$	70.0648;116.0703;84.0445;56.0494;71.0683	4.10	Proline	Amino acid and derivatives
58	5.64	$C_{15}H_{18}O_3$	247.1331	$[M+H]^+$	247.1342;229.1229;183.1174;201.1289;157.1018	0.29	Leucodin	Amino acid and derivatives
59	0.57	$C_{11}H_{17}NO_8$	290.0876	$[M-H]^-$	128.0354;129.0382;291.0124;201.0593;200.0564	1.36	N-Fructosyl pyroglutamate	Amino acid and derivatives
60	1.83	$C_{12}H_{24}O_6$	265.1647	$[M+H]^+$	265.1445;85.0281;193.0870;111.0434;175.0741	1.04	Hexyl glucoside	Glycoside
61	2.18	$C_{17}H_{32}O_{10}$	397.2071	$[M+H]^+$	85.0283;397.1543;97.0280;145.0507;191.1058	0.23	1-Hexanol arabinosylglucoside	Glycoside
62	2.75	$C_{16}H_{22}O_7$	327.1447	$[M+H]^+$	137.0602;163.0755;133.0653;327.1598;295.1355	0.82	1-Methoxy-3-(4-hydroxyphenyl)-2E-propenal 4'-glucoside	Glycoside
63	0.90	$C_{16}H_{18}O_8$	339.1073	$[M+H]^+$	147.0439;119.0496;177.0541;91.0541;145.0288	2.02	Hydrojuglone glucoside	Glycoside
64	3.91	$C_{21}H_{21}O_{10}$	431.0997	$[M-H]^-$	269.0465;431.1011;59.0141;432.1023;270.0509	1.59	Pelargonidin-3-O-glucoside	Glycoside
65	4.18	$C_{21}H_{36}O_{10}$	447.2237	$[M-H]^-$	89.0240;59.0140;447.2194;71.0136;149.0458	0.72	Pentose-Hexose + C10H17	Glycoside
66	2.90	$C_{20}H_{28}O_8$	414.2128	$[M+NH^4]^+$	199.1127;155.0856;129.0699;93.0332;128.0617	0.50	Lobetyolin	Miscellaneous
67	2.91	$C_{11}H_{10}$	143.0856	$[M+H]^+$	143.0858;128.0620;115.0545;155.0600;144.0895	3.06	1-Methylnaphthalene	Miscellaneous
68	4.54	$C_{19}H_{29}NO_9$	416.1913	$[M+H]^+$	254.1382;236.1084;218.1182;205.0855;187.0753;161.0602	0.93	Codonopiloside A	Miscellaneous

69	4.86	$C_{14}H_{22}NO_4$	268.1539	$[M+H]^+$	250.1423;220.1332;205.0860;161.0592	0.87	Codonopyrrolidium B	Miscellaneous
70	9.26	$C_{14}H_{22}O_2$	221.1543	$[M-H]^-$	221.1530;222.1590;206.1304;205.1246;162.0325	3.28	Isokobusone	Miscellaneous
71	0.69	$C_8H_6O_3$	151.0390	$[M+H]^+$	151.0391;152.0703;123.0438;108.0201;121.0281	0.28	4-Hydroxyphthalide	Miscellaneous
